# Differential Gene Expression Associated with Honey Bee Grooming Behavior in Response to *Varroa* Mites

**DOI:** 10.1007/s10519-017-9834-6

**Published:** 2017-02-03

**Authors:** Mollah Md. Hamiduzzaman, Berna Emsen, Greg J. Hunt, Subhashree Subramanyam, Christie E. Williams, Jennifer M. Tsuruda, Ernesto Guzman-Novoa

**Affiliations:** 1 School of Environmental Sciences, University of Guelph, 50 Stone Road East, Guelph, ON N1G 2W1 Canada; 2 Department of Animal Science, Ataturk University, 25240 Erzurum, Turkey; 3 Department of Entomology, Purdue University, 901 West State Street, West Lafayette, IN 47907 USA; 4 Department of Agronomy, Purdue University, 915 West State Street, West Lafayette, IN 47907 USA; 5 Crop Production and Pest Control Research Unit, USDA­ARS, West Lafayette, IN 47907 USA; 6 Clemson University, 130 McGinty Ct, Clemson, SC 29634 USA

**Keywords:** Grooming behavior, *Apis mellifera*, Gene expression, *Varroa destructor*, *Neurexin*, mRNA abundance

## Abstract

Honey bee (*Apis mellifera*) grooming behavior is an important mechanism of resistance against the parasitic mite *Varroa destructor*. This research was conducted to study associations between grooming behavior and the expression of selected immune, neural, detoxification, developmental and health-related genes. Individual bees tested in a laboratory assay for various levels of grooming behavior in response to *V. destructor* were also analyzed for gene expression. Intense groomers (IG) were most efficient in that they needed significantly less time to start grooming and fewer grooming attempts to successfully remove mites from their bodies than did light groomers (LG). In addition, the relative abundance of the *neurexin-1* mRNA, was significantly higher in IG than in LG, no groomers (NG) or control (bees without mite). The abundance of *poly U binding factor kd 68* and *cytochrome p450* mRNAs were significantly higher in IG than in control bees. The abundance of *hymenoptaecin* mRNA was significantly higher in IG than in NG, but it was not different from that of control bees. The abundance of *vitellogenin* mRNA was not changed by grooming activity. However, the abundance of *blue cheese* mRNA was significantly reduced in IG compared to LG or NG, but not to control bees. Efficient removal of mites by IG correlated with different gene expression patterns in bees. These results suggest that the level of grooming behavior may be related to the expression pattern of vital honey bee genes. *Neurexin-1*, in particular, might be useful as a bio-marker for behavioral traits in bees.

## Introduction

The parasitic mite *Varroa destructor* has caused the loss of millions of honey bee (*Apis mellifera*) colonies and thus is considered the number one health problem of honey bees worldwide (Stankus 2008; Guzman-Novoa et al. 2010; Le Conte et al. 2010). Varroa mites weaken bees by feeding on their haemolymph after wounding their cuticle, which may result in the invasion of secondary pathogens, leading to their early death (De Jong et al. 1982). *Varroa* mites also suppress bee immunity (Yang and Cox-Foster 2005; Navajas et al. 2008; Nazzi et al. 2012) and act as vectors of several honey bee viruses (Kevan et al. 2006; Emsen et al. 2015; Hamiduzzaman et al. 2015; Anguiano-Baez et al. 2016). On the behavioral level, *Varroa* hampers non-associative learning (Kralj et al. 2007), and reduces the proportion of foragers that return to the hive (Kralj and Fuchs 2006). Control of *Varroa* infestations in honey bee colonies has become a daunting task for beekeepers and scientists. Most beekeepers use synthetic miticides to control the parasites, but the continuous use of pesticides leads to the development of resistance in the mites (Milani 1999). Furthermore, the use of pesticides increases the risk of contamination of honey and other hive products (Wallner 1999). Other ways of controlling this mite are thus needed. One potential approach to controlling *V. destructor* would be the development of honey bee strains resistant to the parasite. This could theoretically be achieved by natural selection (bees not treated against the mite) or by breeding bees expressing traits associated to mite resistance or tolerance (Rinderer et al. 2010; Arechavaleta-Velasco et al. 2012; Guzman-Novoa et al. 2012; Hunt et al. 2016).

The original host of *V. destructor*, the Asiatic bee *Apis cerana*, naturally resists infestations by *Varroa* through multiple mechanisms. The most important mechanism of *A. cerana* resistance appears to be through grooming behavior (Peng et al. 1987). The western honey bee, *A. mellifera*, also expresses grooming behavior against *Varroa*, but to a lesser degree than its Asiatic counterpart (Buchler et al. 1992; Fries et al. 1996). Through grooming behavior, some adult bees physically remove mites from their bodies using their legs and mandibles (Ruttner and Hanel 1992; Fries et al. 1996; Boecking and Spivak 1999; Bahreini and Currie 2015). Grooming behavior is also a defense mechanism against tracheal mites (Pettis and Pankiw 1998; Danka and Villa 2003, 2005).

Bees groom themselves at various levels of intensity. Guzman-Novoa et al. (2012) reported that bees that groom at high intensity remove significantly more mites from their bodies than bees that do it lightly, suggesting that grooming intensity is an important factor for resistance to *Varroa*. Not much is known about the genetic mechanisms regulating grooming behavior but it appears to be a quantitative trait with a genetic component (Moretto et al. 1993; Page and Guzman-Novoa 1997; Arechavaleta-Velasco et al. 2012). Grooming behavior is also influenced by environmental effects (Currie and Tahmasbi 2008). The degree to which grooming behavior is influenced by genes is unknown but, if there is significant genetic variability for this trait, bees could be bred for high grooming expression and intensity to develop resistant stock to *V. destructor* (Hunt et al. 2016).

A number of studies have shown that *V. destructor* parasitism alters the expression pattern of immune-related (Yang and Cox-Foster 2005; Navajas et al. 2008; Hamiduzzaman et al. 2012) and behavioral-related genes in honey bees (Le Conte et al. 2011). However, there are no studies of gene expression in bees that exhibit intense grooming behavior. To learn more about genes that may be involved in bee behavioral mechanisms of resistance against mites, we explored the association of different degrees of grooming behavior with mRNA abundance of some candidate genes for which expression information exists for other traits, and from some genes tested for the first time. We chose genes that have reduced expression in response to *V. destructor* parasitism such as the immune related gene, *hymenoptaecin* (*Hym*), the putative cell proliferation regulator, *poly U binding factor kd 68* (*pUf68*), and a gene related to longevity, development and general health, *vitellogenin* (*Vg*). We also tested a gene for the autophagy-linked FYVE protein, *blue cheese* (*BlCh*), whose expression is changed by *V. destructor* parasitism (Yang and Cox-Foster 2005; Navajas et al. 2008; Dainat et al. 2012; Hamiduzzaman et al. 2012). Honey bees like other insects rely on detoxification genes such as the cytochrome p450 gene, *CYP9Q3*, which has shown altered expression patterns when insects are exposed to different types of chemicals (Mao et al. 2011), or when performing physical activities such as hygienic behavior (Boutin et al. 2015). But the expression of *CYP9Q3* has not been assessed for bees that are exposed to mites or as a response to other behavioral activities such as grooming behavior. Expression of the neural gene *neurexin-1* (*AmNrx1*) occurs primarily in the central nervous system and in the mushroom body of the brain, which is an important organ for higher-order processing and learning in the bee (Heisenberg 1998; Szyska et al. 2008) and *AmNrx1* is among a small number of candidate genes for honey bee grooming behavior identified in a quantitative trait locus for honey bee grooming behavior (Arechavaleta-Velasco et al. 2012). *AmNrx1* is also known to be related to autism disorder in humans, a syndrome that is associated with repetitive movements or ataxias (Feng et al. 2006; Sudhof 2008; Reichelt et al. 2012) and in self-grooming behavior in mice (Etherton et al. 2009). Therefore this gene could potentially affect grooming behavior, but has not been studied in relation to this trait in bees.

The objectives of this study were (1) to correlate the effect of two levels of grooming behavior (light and intense) with the time required to start grooming and with the number of attempts needed by individual bees exposed to *Varroa* mites to successfully remove the parasite from their bodies, and (2) to analyze the association between these levels of grooming behavior and the expression of selected genes in tested bees.

## Materials and Methods

### Collection of *V. destructor* Mites

Grooming experiments were conducted at the Honey Bee Research Centre of the University of Guelph, in Guelph, Ontario, Canada between April and August, 2013. Adult foundress *Varroa* mites from heavily infested honey bee colonies were harvested from brood cells containing white-eyed pupae using a fine paint brush. The harvested mites were held in Petri dishes lined with moist filter paper and containing two white-eyed bee pupae collected from a non-infested colony; the pupae served as a food source for the mites. The mites were kept at room temperature (26 ± 2 °C) and used within 2 h from the time of collection.

### Grooming Behavior in Individual Bees

Grooming behavior at the individual level was performed in the laboratory using a modified version of the method described by Aumeier (2000). All worker bees were sampled from five local, randomly selected colonies, presumably representing a broad sample of genotypes because queens of the colonies were open mated to approximately 12–20 haploid drones. Worker bees for all treatments were collected from the brood nest of the source colonies using a bee vacuum (Gary and Lorenzen 1990). Individual Petri dishes (9 cm diameter) were prepared in advance of the assays by lining their bottom with a circular piece of white filter paper to provide contrast for observation of bees and mites. Petri dishes were covered with plastic wrap. The plastic wrap was perforated 20–30 times with a nail (50 × 3 mm) in order to allow air to pass through. One worker bee was introduced into each dish and was then given 2–3 min to become accustomed to the Petri dish. The plastic wrap was then lifted slightly in order to place a single mite on the bee’s thorax using a fine brush (except for control bees that were only touched with the brush on the thorax). A stopwatch was started immediately upon application of the mite and the bee was observed for up to 3 min. Grooming instances exhibited by the bee were recorded, specifically describing the time elapsed until she started to groom, the number of grooming attempts, whether or not she removed the mite and the intensity with which she groomed. The following variables were recorded: time (s) elapsed from the moment a mite was placed on the bee thorax until she started to groom, time to mite removal, and the number of grooming attempts a bee required to successfully remove a mite. A grooming attempt was defined as an uninterrupted period of time during which grooming was observed, and that ended when the bee paused (a bee could have several of these grooming instances within 3 min). In the event that a bee successfully removed the mite within 3 min, the trial ended and the time of removal was recorded. Bees that could not remove the mite within 3 min were only classified by the intensity with which they groomed. “Light grooming” (LG) consisted of slow swipes of one or occasionally two legs across the thorax or abdomen. “Intense grooming” (IG) consisted of vigorous wiping and shaking and always involved the use of more than two legs. Whether the grooming was recorded as “light” or “intense” and how many grooming attempts were performed by each bee was left to the observer’s judgement. However, there was only one observer, and therefore all incidences were judged by the same person as described by Guzman-Novoa et al. (2012). Some bees did not groom and were recorded as “no grooming” (NG). Since control bees were not exposed to the irritation caused by *Varroa* mites and were not assessed for mite removal, they were only evaluated for whether or not they groomed, and for those that groomed, the time to start grooming and grooming attempts within 3 min were recorded. Grooming trials were performed with a total of 240 bees. Samples of 12–16 individuals for each IG, LG, NG and control bees were randomly collected at the end of trials and frozen at −70 °C for further analysis of gene expression.

### RNA Extraction and cDNA Synthesis

Total RNA was extracted by homogenizing each adult bee sample in extraction buffer as per Chen et al. (2000). The homogenates were extracted twice with chloroform and the RNA was precipitated using LiCl as described by Sambrook et al. (1989). The amount of total RNA extracted was determined with a spectrophotometer (Nanovue GE Healthcare, Cambridge, UK). RNA samples were stored at−70 °C. For cDNA synthesis, 2 µg of total RNA was reverse-transcribed using Oligo (dT)_18_ and M-MuLV RT with the RevertAid™ H Minus First Strand cDNA Synthesis Kit (Fermentas Life Sciences, Burlington, ON, Canada), following the instructions of the manufacturer. The cDNA was stored at −20 °C.

### Primers

The primers used to amplify the genes evaluated are shown in Table 1. To design some of the primers, the complete sequences of the genes were obtained from the National Centre for Biotechnology Information (NCBI) (http://www.ncbi.nlm.nih.gov). The sequences were aligned using CLUSTALX and the primers were designed using the Gene Runner (Version 3.05, Hastings Software, Inc., NY). The oligo nucleotides were ordered from Laboratory Services of the University of Guelph (Guelph, ON, Canada).

**Table 1:** Primers used for amplification of the target and constitutive control genes

Gene name	Primer sequence (5′–3′)	Gene ID	Band size	References
Hym*	F: CTCTTCTGTGCCGTTGCATA R: GCGTCTCCTGTCATTCCATT	GB17538	200 bp	Evans (2006)
pUf68*	F: CAAGACCTCCAACTAGCATG R: CAACAGGTGGTGGTGGTG	GB13651	201 bp	Hamiduzzaman et al. (2012)
BlCh*	F: GTGCTTGGGTTAGGATGTGTAC R: GTTAATCTTCTTCCGCTACTG	GB10249	218 bp	Hamiduzzaman et al. (2012)
AmNrx1*	F: ACGCCCACCACAGAGATGAC R: CATTTGGATCCTGGCAGAAG	FJ580046	259 bp	This study
CYP9Q3*	F: GTTCCGGGAAAATGACTAC R: ACTCTCGACGCACATCCTG	XM_006562300	296 bp	Mao et al. (2011) This study
Vg*	F: CTGTCGATGGAGAAGGGAACT R: CTTGCCTACGAGTCTTGCTGT	NM_001011578	370 bp	This study
RpS5**	F: AATTATTTGGTCGCTGGAATTG R: TAACGTCCAGCAGAATGTGGTA	GB11132	115 bp	Evans (2006)
GAPD2**	F: GATGCACCCATGTTTGTTTG R: TTTGCAGAAGGTGCATCAAC	GB14798	203 bp	Thompson et al. (2007)
AmNrxn1* (qRT-PCR)	F: ACGCCCACCACAGAGATGAC R: CCGATTATTAAGGCAGCGTTCT	FJ580046	137 bp	This study
AmRPL8** (qRT-PCR)	F: TGGATGTTCAACAGGGTTCATA R: CCGATTATTAAGGCAGCGTTCT		122 bp	This study

*F* forward primer, *R* reverse primer

*Target

**Constitutive control genes

### PCR Amplifications

Each of the target genes (except *CYP9Q3*) was co-amplified together with the honey bee ribosomal protein *RpS5* gene (Thompson et al. 2007) in the same tube and reaction as a constitutive control. The *glyceraldehyde 3-phosphate dehydrogenase2* (*GAPD2*) gene (Thompson et al. 2007) was used as another standard control to co-amplify with *CYP9Q3*. All PCR reactions were done with a Mastercycler (Eppendorf, Mississauga, ON, Canada). Each 15 µL of reaction contained 1.5 µL of 10× PCR buffer (New England BioLabs, Pickering, ON, Canada), 0.5 µL 10 mM of dNTPs (Bio Basic Inc., Markham, ON, Canada), 1 µL of 10 µM for each primer of target and housekeeping genes (Laboratory Services, University of Guelph, Guelph, ON, Canada), 0.2 µL 5 U/µL of Taq polymerase (New England BioLabs, Pickering, ON, Canada), 2 µL of the cDNA sample, and 6.8 µL of dd H_2_O. To amplify *AmNrx1, CYP9Q3, Hym* and *Vg*, the thermocycler was programmed to run at 94 °C for 3 min, followed by 35 cycles of 30 s at 94 °C, 60 s at 58 °C and 60 s at 72 °C, and a final extension step at 72 °C for 10 min. To amplify *pUf68* and *BlCh*, the annealing temperature was 55 °C while the other conditions described above remained the same.

### Separation and Semi-Quantification of PCR Products

PCR products were separated on 1% TAE agarose gels and stained with ethidium bromide. A 100 bp DNA ladder (Bio Basic Inc., Markham, ON, Canada) was included in each gel. Images of the gels were captured using a digital camera with a Benchtop UV Transilluminator (BioDoc-It^M^ Imaging System, Upland, CA). The intensity of the amplified bands was measured in pixels using the Scion Image Program (Scion Corporation, Frederick, MD) as per Dean et al. (2002). The ratio of band intensity between the target gene and the housekeeping gene was calculated to determine the relative expression units (REU) of each gene. To determine whether quantification at 35 amplification cycles was not affected by signal saturation of the band intensities, randomly selected samples with high, medium and low REUs were also quantified in the same manner with fewer amplification cycles, and the pattern of expression based on the REU values were not significantly different when 25, 30 and 35 amplification cycles were used (F_2,15_ = 0.30, p = 0.75). We analyzed results at 35 cycles because in most cases the relationship between the number of cycles and molecules is relatively linear at 35 cycles when semi-quantitative RT-PCR is used, which provides high amplification efficiency.

### Quantitative Real-Time-PCR Methods

To confirm the correlation between *AmNrx1* mRNA abundance and grooming behavior obtained with the semi-quantification method (this gene was the gene that most consistently correlated with grooming behavior), target-specific qRT-PCR primers (Table 1) corresponding to the *Neurexin1A* gene were designed using the Primer Express 3.0 software (ABI, Applied Biosystems, Foster City, CA). The qRT-PCR was performed using the Light Cycler 480 II Real Time PCR System (Roche, Indianapolis, IN) using the SYBR Green dye-based detection system. All reactions were performed in a final volume of 10 µL, consisting of 5 µL of SensiFAST SYBR no-ROX master mix (Bioline, Taunton, MA), gene-specific primers at a final concentration of 0.2 µM each, and 20 ng of cDNA template. No-template and no-reverse transcriptase samples were included in each PCR plate as negative controls. Along with the target-gene, the qRT-PCR plate also included *AmRPL8* (60S ribosomal protein L8) as an internal reference housekeeping gene to verify equal amounts of target cDNA in all samples. All reactions were set up in triplicate for each of the biological replicates. PCR conditions were as follows: 95 °C for 5 min, 45 cycles of 95 °C for 10 s, 60 °C for 20 s, and 72 °C for 30 s. To determine the specificity of the reaction a melt curve analysis was carried out following PCR, confirming amplification of a single product. Quantification of gene expression, displayed as Relative Expression Value (REV) was calculated using the Relative Standard Curve Method (User Bulletin 2: ABI PRISM 7700 Sequence Detection System) as described in Subramanyam et al. (2006).

### Statistical Analysis

Data on time to start grooming, number of grooming attempts, time to successful mite removal and gene expression were subjected to analysis of variance (ANOVA), excluding non-groomers and negative control values from the analyses because they represented 0 values. A correlation analysis was performed with *AmNrx1*expression data from the semi-quantification method and from the qRT-PCR to validate results. To obtain descriptive statistics and perform ANOVAS, the package IBM-SPSS v. 23 (SPSS Inc., Chicago, IL) was used. Significant differences among means were separated with Fisher’s protected LSD or Tamhane’s T2 tests (α = 0.05).

## Results

**Fig. 1:**
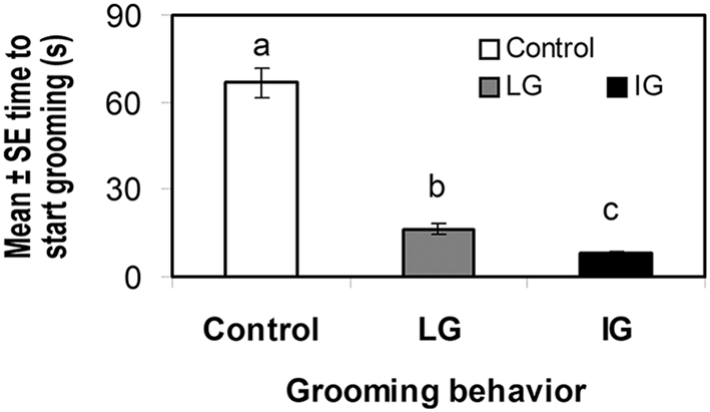
Mean time to start grooming ± SE (s) within 3 min in individual worker bees either not exposed to *V. destructor* (control bees, only touched with a fine brush on the thorax), or exposed to a mite (by placing a mite on their bodies). Exposed bees responded by not grooming (excluded from the analysis due to 0 values), or by grooming at light pace (LG) or at vigorous pace (IG). *Different letters* indicate significant differences of means based on analysis of variance and Tamhane’s T2 tests (p < 0.01; n = 240)

**Fig. 2:**
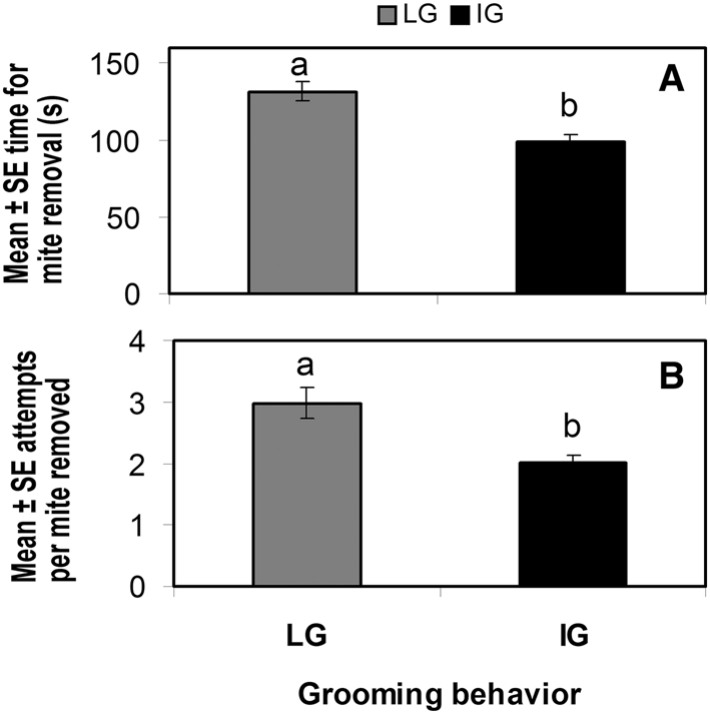
Mite removal success of worker bees exposed to *V. destructor* for 3 min in the laboratory. **a** Mean time spent for mite removal ± SE (s) and **b** mean number of attempts until successful mite removal for individual bees exposed to *V. destructor* by placing a mite on their bodies. Only bees that responded by grooming at light pace (LG) or at vigorous pace (IG) were included in the analysis. *Different letters* indicate significant differences of means based on analysis of variance and Tamhane’s T2 tests (p < 0.01; n = 210)

IG bees started to groom themselves significantly faster than LG and control bees. LG bees also initiated grooming activity significantly faster than control bees (F_3, 206_ = 220.83, p < 0.0001), whereas NG did not groom at all within the 3 min lapse of the trial (Fig. 1). To achieve mite removal success, IG bees required significantly less time and fewer grooming attempts than LG bees (F_2, 177_ = 76.50, p < 0.0001 and F_2, 207_ = 50.65, p < 0.0001 for time and removal attempts, respectively), whereas NG bees did not groom or remove mites (Fig. 2a, b), indicating that IG bees are more efficient at removing mites than other bees.

**Fig. 3:**
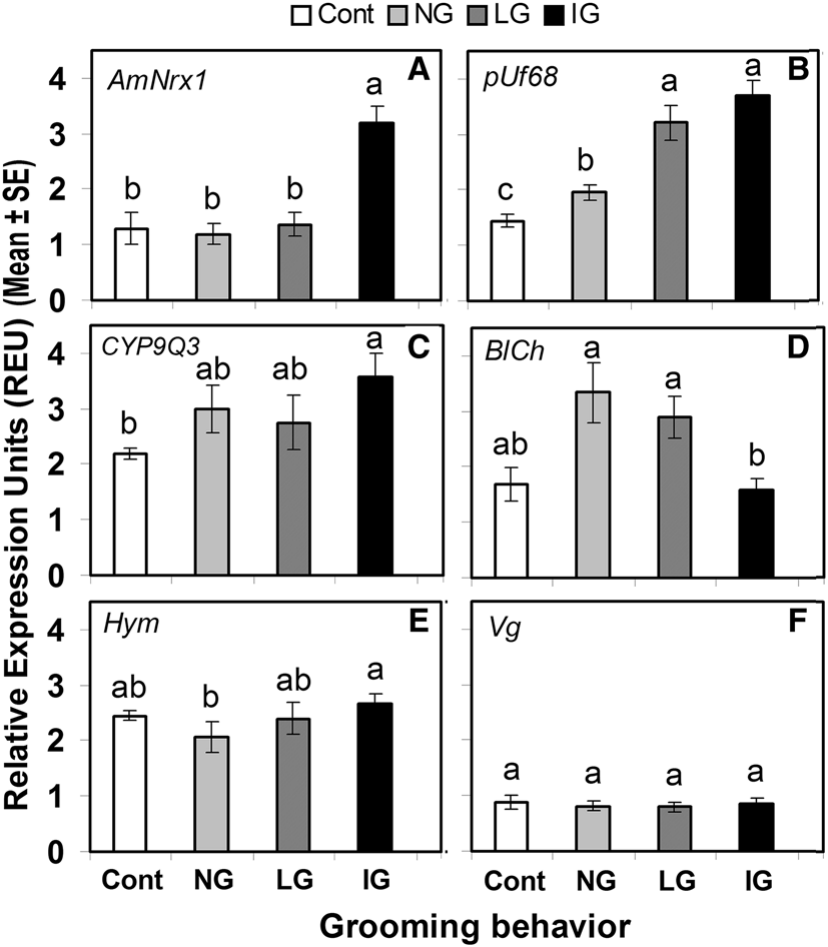
Relative RT-PCR quantification units of *AmNrx1* (**a**), *pUf68* (**b**), *CYP9Q3* (**c**), *BlCh* (**d**), *Hym* (**e**) and *Vg* (**f**), relative to house-keeping genes (*RpS5* or *GAPD2*) of individual worker bees not exposed to *V. destructor* (control bees, only touched with a fine brush on the thorax) or exposed to it (by placing a mite on their bodies). Exposed bees responded by not grooming (NG), or by grooming at light pace (LG) or at vigorous pace (IG). *Different letters* indicate significant differences of means based on analysis of variance and Fisher’s protected LSD tests (p < 0.05; n = 64 for all genes, except for *AmNrx1* with n = 52 and *Hym* with n = 40)

The expression of *AmNrx1* was significantly higher in IG than in LG, NG and control bees. There were no significant differences in the level of expression of this gene among LG, NG and control bees, indicating that only intense grooming was associated with a high expression level of *AmNrx1* (F_3, 48_ = 12.20, p < 0.0001, Fig. 3a).

The expression of *pUf68* increased significantly in both IG and LG bees relative to NG and control bees with no differences between IG and LG bees. However, the level of gene expression in NG was higher than in control bees (F_3, 60_ = 20.94, p < 0.0001, Fig. 3b).

The expression of *CYP9Q3* was significantly higher in IG than in control bees, but not different from that of NG and LG bees (F_3, 60_ = 5.04, p < 0.01, Fig. 3c). Conversely to the above results, the expression of *BlCh* was significantly higher in LG and NG than in IG bees, while there were no significant differences in expression of *BlCh* gene between control bees and bees of the rest of the treatments (F_3, 60_ = 5.45, p < 0.05, Fig. 3d). *Hym* was significantly upregulated in IG compared to NG bees, but there were no significant differences in gene expression levels among NG, LG and control bees (F_3, 36_ = 4.12, p < 0.05, Fig. 3e). Finally, expression of *Vg* was not associated to grooming behavior or the presence of *Varroa*, since no differences in expression for this gene were observed among all treatments (F_3, 60_ = 0.125, p > 0.05, Fig. 3f).

The results from qRT-PCR of *AmNrx1* supported those obtained with the semi-quantitative method. IG bees had higher *AmNrx1* mRNA abundance than did LG and NG bees (F_2, 22_ = 3.768, p < 0.05). Additionally, expression data from the semi-quantification method and from the qRT-PCR for the same bees were significantly correlated (r = 0.65, p < 0.001).

## Discussion

Bees that performed instances of intense grooming were significantly faster to start grooming and required fewer grooming attempts and less time to remove *Varroa* mites from their bodies than bees performing light grooming. These results indicated that IG bees were very sensitive to the mite presence on their bodies and were efficient at removing them. Guzman-Novoa et al. (2012) compared different presumably *Varroa*-susceptible and resistant genotypes of honey bees for grooming ability, and found that a significantly higher number of mites were dislodged from the bees’ bodies by intense grooming than by light grooming regardless of genotype, which agrees with the findings here reported.

Grooming behavior allows insects to clean their body surface and sensory organs (Zhukovskaya et al. 2013). Therefore, this behavior is linked with the ability of the insect to perceive stimuli from its environment. Parasitic mites provide mechanical and chemosensory stimuli, which may result in the initiation of grooming behavior by the affected bee. Thus, sensory recognition of the parasite could lead to behavioral and immune responses such as grooming behavior (Roode and Lefevre 2012). More efficient grooming bees may rely on quick recognition of *Varroa* presence by tactile or chemosensory sensors. This in turn would activate defense mechanisms, including reacting through physical activities such as grooming behavior, to successfully remove the mites from their bodies. The age and reproductive status of mites could also be a factor that influences the sensitivity of honey bees to perform grooming behavior. Kirrane et al. (2012) evaluated in laboratory cages the grooming response of honey bees to *V. destructor*, and concluded that the grooming success of bees was affected by the age and reproductive status of the mites. The highest mite drop was for daughter mites and the lowest for foundress mites, which suggests that the former age group stimulated bees to remove mites from their bodies more frequently than when parasitized with foundresses. We used foundress mites in our study, so, perhaps had we used only daughter mites we would have seen a higher frequency of mite removal and probably higher levels of gene expression. This hypothesis however, remains to be tested.

Supporting the above potential explanations, Biswas et al. (2010) reported that the expression of the neural gene *AmNrx1* was affected by sensory experience in honey bees, which may play a role in the development of synaptic connections that could influence learning and the expression of behavioral traits. Also, Arechavaleta-Velasco et al. (2012) demonstrated that some candidate genes, including *AmNrx1*, were associated with grooming behavior. Similarly, successful mite removal by IG bees in this study suggested that these bees may have a higher sensitivity to *Varroa*, resulting in increased expression of neuron-related genes, such as *AmNrx1*. The significantly higher expression level of *AmNrx1* in IG than in LG, NG and control bees supported results from the above studies and the notion that this gene is associated with grooming behavior and/or physical activity. Further study is needed to distinguish between *AmNrx1* effects on grooming or activity states.

The putative cell proliferation regulator protein, *pUf68*, also known as *half pint*, plays important regulatory roles in controlling the production of complex diverse proteins in a wide range of organisms (Bourgeois et al. 2004). *pUf68* is particularly known for its role in pre-mRNA splicing, which could possibly be related to physical activity in the insect. It might be that products of *pUf68* are linked to functions of the peripheral nervous system (PNS) of bees. Physical activity such as grooming behavior in bees might have an impact on the splicing of *pUf68* and transcript proliferation in cells through the PNS. The significantly higher expression of *pUf68* in both IG and LG than in NG and control bees suggested that it could be affected by grooming activity or vice versa. Contrary to our results, the expression of *pUf68* was found to be suppressed by *Varroa* parasitism in adult bees (Yang and Cox Foster 2005; Navajas et al. 2008) and brood (Dainat et al. 2012; Hamiduzzaman et al. 2012). Perhaps the difference between our results and those of the above studies is related to time of exposure to the mite. In our study, bees were exposed to *Varroa* <3 min and so, presumably the mite did not have time to inoculate immune-suppressive effectors through its saliva while feeding on the bees’ haemolymph (Yang and Cox-Foster 2007; Richards et al. 2011). Therefore, the mite may have been unable to suppress the expression of this immune related gene in the bees. Probably the high physical activity of grooming bees, leads to physiological changes resulting in higher expression of *pUf68*. It is also possible that the expression of this gene unchains higher physical activity through neural mechanisms stimulated by the presence of a mite. Regardless of why the expression of this gene is affected, this is the first report of a relationship between *pUf68* mRNA abundance and grooming behavior in bees. Further studies will be needed to clarify the mechanisms through which grooming activity and the expression of this gene in honey bees are related.

Expression of the detoxification gene, *CYP9Q3*, in IG bees was significantly higher than in control bees, but similar to that of LG and NG bees. These results suggested an effect on gene expression related to the presence of *Varroa* on the bee’s body (since control bees were treated identically but not challenged with a mite) but not necessarily associated with the physical activity of grooming behavior. It may be that the short exposure to the mite unchains a physiological reaction leading to a higher expression of this gene only in bees exposed to the mite regardless of their physical activity. Perhaps expression of *CYP9Q3* can respond to a non-chemical stress, such as the attachment of a *Varroa* mite (Mao et al. 2011; Boncristiani et al. 2012). Supporting the hypothesis that *CYP9Q3* is not related to physical activity, Boutin et al. (2015) found that *cytochrome p450* genes were over-expressed in non-hygienic bees compared to hygienic bees, and hypothesized that the products of these genes degrade the odorant pheromones and chemicals that signal the presence of diseased brood and thus resulted in these bees being less efficient in detecting killed brood. Although no studies have been conducted to demonstrate a relationship between mite odors and grooming behavior, it is possible that the increased expression of *CYP9Q3* in our study had been influenced by scents of the mite. Odorant substances such as pheromones may influence gene expression in the honey bee. For example, Grozinger et al. (2003) reported that queen mandibular pheromone (QMP) affects gene expression in the bee brain, which showed correlation with behavioral responses (i.e. brood care, nursing) in adult worker bees.

Navajas et al. (2008) reported that the expression of the autophagy-linked gene *BlCh*, was up-regulated in bees presumed to be *Varroa*-tolerant, while the expression of *Dlic2* and *Atg18* genes, which influence neural reactions, was down-regulated. Interestingly, in another study, the expression of *BlCh* was negatively correlated with *Dlic2* and *Atg18* in *Varroa*-parasitized bees (Simonsen et al. 2007). These findings agree with our results of increased *BlCh* expression in NG and LG bees and of decreased expression of this gene in IG bees. Intense physical activity during grooming could be related to the nervous system being stimulated by the products of *Dlic2* and *Atg18* genes, which would also result in suppression of *BlCh* in IG bees. Future experiments however, are required to confirm whether this explanation is plausible.

The expression of *Hym* in IG bees was similar to that of LG and control bees, but it was lower in NG bees. This result is difficult to explain but perhaps it is related to differences in activity between the groups of bees. Control bees as well as LG and IG bees all groomed (and thus were active), whereas NG bees showed reduced activity. It also seems that mite parasitism had no effect on *Hym* expression since control bees were not exposed to mites but did not differ from LG and IG bees that were parasitized by a mite. Another possibility is that mRNA abundance of genes such as *Hym, CYP9Q3* and *AmNrx1* are all increased by stress, which in turn increases the tendency for intense grooming. Genotypic variation between bees of different sources could also differentially influence gene expression in *Varroa*-parasitized and not parasitized bees (Navajas et al. 2008). However, these and other potential explanations of our results, require further experimentation.

There was no significant difference in the expression of the developmental and general health related gene, *Vg*, among bees of the different treatments, indicating that neither physical activity nor short exposure to *Varroa* affects the expression of this gene and that this gene does not seem to be related to grooming behavior.

Because *Varroa* poses a serious threat to bee health, researchers have been trying to find mite-resistance traits in bees. Several studies have indicated that grooming behavior may be a very important trait in conferring resistance to bees against the mite at the colony and individual levels (Moretto et al. 1993; Arechavaleta-Velasco and Guzman-Novoa 2001; Andino and Hunt 2011; Hunt et al. 2016; Invernizzi et al. 2016). These and a previous study (Guzman-Novoa et al. 2012) demonstrate and confirm the importance of efficient grooming for successful mite removal in honey bees. At the molecular level, Arechavaleta-Velasco et al. (2012) searched for genes influencing grooming behavior by analyzing the DNA of bee genotypes in backcross workers derived from high- and low-grooming parents. These workers varied in tendency to initiate grooming instances after being challenged with *Varroa* mites on their bodies. These researchers identified a single chromosomal region containing a set of candidate genes, which includes *AmNrx1*, using quantitative-trait-locus (QTL) interval mapping. Consistent with this finding, of all the genes tested in this study, *AmNrx1* was most highly and consistently related to intense grooming and thus, warrants further investigation.

One limitation of this study is the small number of genes selected to study in the context of grooming behavior. Analyzing more genes based on their specific function might have been more informative in evaluating their expression pattern during grooming instances. Despite this limitation, some of the selected genes showed association with IG, indicating that probably multiple genes rather than a single gene might be involved in regulating grooming behavior. However, whether the genes are influencing the behavior or vice versa still needs to be confirmed. Therefore, more studies need to be conducted to understand the involvement of some of these and other genes that are related to neural sensitivity as they respond to the irritation caused by ectoparasitic mites on the bees. Finding candidate genes that influence the intensity with which bees groom themselves in response to parasitic mites is critical for developing marker assisted selection assays to breed for mite resistance in honey bees.
